# The association between relative deprivation and cyber-ostracism: the mediating role of social emotions and the moderating role of self-esteem

**DOI:** 10.3389/fpsyg.2026.1834878

**Published:** 2026-08-06

**Authors:** Xiaokang Lin, Xiwen Yuan, Yunlong Tian

**Affiliations:** 1 School of Teacher Education, Guangzhou Huashang College, Guangzhou, China; 2 Research Base of Guangdong Basic Education Development, Guangzhou Huashang College, Guangzhou, China; 3 Student Affairs Division, Guangzhou Huashang College, Guangzhou, China; 4 School of Educational Science, Huangshan University, Huangshan, China

**Keywords:** cyber-ostracism, moderated mediation, online interaction, relative deprivation, self-esteem, social emotion

## Abstract

As everyday social interaction increasingly unfolds online, individuals who feel relatively deprived may turn to these platforms for connection yet become more exposed to cyber-ostracism. However, the socioemotional mechanisms and individual resources that bound this association remain insufficiently understood. To address this gap, the present study tested a moderated dual mediation model to investigate the mediating roles of positive and negative social emotions in the relationship between relative deprivation and cyber-ostracism, as well as the moderating effect of self-esteem. A total of 1,508 undergraduate students (43.3% female; M age = 19.43 years) completed validated measures of relative deprivation, social emotion, self-esteem, and cyber-ostracism. The parallel mediation model showed a positive association between relative deprivation and cyber-ostracism, with significant indirect effects through reduced positive social emotion [indirect effect = 0.03, 95% CI = (0.011, 0.039), 6.78% of total effect] and elevated negative social emotion [indirect effect = 0.04, 95% CI = (0.012, 0.069), 10.82% of total effect]. In the full moderated mediation model, self-esteem moderated the paths from relative deprivation to positive social emotion, negative social emotion, and cyber-ostracism; the moderated indirect effect via positive social emotion was significant, whereas the path from negative social emotion to cyber-ostracism was not significant in this model. These findings advance theoretical understanding of the emotional pathways underlying cyber-ostracism, underscore the protective function of self-esteem, and inform intervention strategies aimed at mitigating deprivation-related risks and fostering healthier online social interactions.

## 1 Introduction

Online interactions such as liking, commenting, and posting have become an increasingly important arena for social exchange and self-evaluation (Hall and Liu, 2022). However, interaction frequency does not necessarily translate into relational closeness; it can also enable new forms of isolation, such as cyber-ostracism. *Cyber-ostracism* refers to individuals’ experiences of being intentionally or unintentionally ignored or excluded in online interactions (Schneider et al., 2017). Prior research has demonstrated that even brief experiences of cyber-ostracism, or even subtle cues of exclusion, can undermine a person’s fundamental needs for belonging and meaningful existence (Williams, 2007; 2008). When such experiences persist over time, they can lead to heightened psychological distress, including depressive symptoms, diminished self-worth, and increased social withdrawal (Li et al., 2022; Williams and Nida, 2022). Beyond internal distress, cyber-ostracism is linked to externalizing behaviors, such as aggression and self-injury (Ding et al., 2022; Shi et al., 2022; Schneider et al., 2017; Liu et al., 2018). Therefore, exploring the mechanism and protecting factors of cyber-ostracism is of great significance.

In the past, people have shared daily life experiences through face-to-face interaction, but with the rapid development of the internet, individuals can now learn about others’ lives and achievements quickly through online interactions (Whiting and Williams, 2013). Although online interaction can facilitate social connection, it also exposes individuals to others’ curated successes and may foster relative deprivation—a subjective sense of being worse off than relevant others that does not necessarily reflect objective standing (Xia and Ma, 2020; Yuan et al., 2025). General strain theory suggests that perceived disadvantage may evoke strain and negative affect, which can be expressed through maladaptive interpersonal responses (Agnew, 2015). Prior research has also suggested that individuals with lower life satisfaction, greater emotional distress, and more frequent negative emotions are more likely to be ostracized (Zhang and Shi, 2017). Together, these findings point to a strong link between relative deprivation and social exclusion. However, given that cyber-ostracism represents a new form of exclusion, whether relative deprivation operates similarly in predicting cyber-ostracism as it does in offline contexts warrants further investigation. Emerging evidence further indicates that relative deprivation may undermine the broader online climate by widening perceived interpersonal distance (Zhu et al., 2024). Accordingly, relative deprivation may be an important antecedent of cyber-ostracism, and clarifying this association is critical for informing strategies that mitigate its adverse consequences and foster more supportive online environments.

### 1.1 Relative deprivation and cyber-ostracism

*Relative deprivation* refers to a subjective cognitive and emotional experience in which individuals perceive their own disadvantages through comparisons with a reference group, consequently leading to experiences of negative emotions, such as anger and dissatisfaction (Crosby, 1976; Smith and Huo, 2014; Smith et al., 2012). Such experiences often arise from unfavorable upward comparisons (Yuan et al., 2025). From the perspective of general strain theory, relative deprivation can be understood as a strain experience because it signals unfair disadvantage, blocked social standing, and threatened belonging. Such strain may elicit negative affect and increase the likelihood of maladaptive interpersonal responses (Agnew, 2015). Prior studies have linked relative deprivation to aggression and online aggression (Greitemeyer and Sagioglou, 2017; Wang et al., 2023; Zhu et al., 2024), as well as ostracism (Jiang and Chen, 2020).

In online contexts, deprivation-related strain may be intensified because social media make others’ achievements and social feedback highly visible (Whiting and Williams, 2013; Yan et al., 2025). Individuals who feel relatively deprived may cope with this strain through hostile, defensive, or withdrawn online interaction patterns (Greitemeyer and Sagioglou, 2019b; Tao et al., 2023; Wang et al., 2023). These responses may weaken reciprocity and make continued interaction more burdensome for others. Consistent with this view, ostracism research suggests that people are more likely to exclude targets who are perceived as norm-violating or less valuable for ongoing interaction (Rudert et al., 2020, 2023). In digital settings, such disengagement may require relatively little interpersonal effort because exclusion can be enacted through reduced feedback, nonresponse, or selective avoidance rather than direct confrontation (Schneider et al., 2017; Zhu and Skoric, 2021). Under these conditions, relative deprivation may shape online interaction patterns in ways that increase the likelihood of being ignored or excluded online. Accordingly, we propose the following hypothesis:

*H1*: Relative deprivation is positively associated with cyber-ostracism.

### 1.2 The mediating role of social emotions

Beyond its cognitive roots, relative deprivation functions as a potent socioemotional stressor. Classical theories of relative deprivation posit that perceived disadvantage elicits intense affective reactions, such as resentment and frustration, which in turn, serve as motivational signals shaping social interpretations and subsequent behaviors (Mummendey et al., 1999; Smith et al., 2012). These reactions are considered *social emotions*, defined as affective experiences elicited by individuals’ evaluative attitudes toward social contexts or events and are grounded in their understanding of their relationships with in-group and out-group members (Hareli and Parkinson, 2008). Accordingly, social emotion may link relative deprivation to the experience of cyber-ostracism.

Prior research on social emotions has primarily treated the construct as unitary or emphasized its negative dimension, leaving open how the positive dimension shapes interpersonal and online behavior (van Kleef and Côté, 2022; Xing and Kuo, 2024). This emphasis provides an incomplete account because social emotion comprises two dimensions: positive social emotion and negative social emotion (Yang et al., 2023). Positive social emotion refers to an engaged and constructive affective orientation toward society and social others, whereas negative social emotion refers to disengaged, dissatisfied, anxious, or restless responses to social conditions. Functionally, positive social emotions foster prosocial behavior and the maintenance of harmonious interpersonal relationships (Sels et al., 2021; Xu et al., 2023), whereas negative social emotions impair interpersonal connection and social adjustment (van Kleef and Lelieveld, 2022). Prior research suggests that positive and negative emotions can co-occur in complex social situations because individuals may respond to multiple and sometimes competing social meanings (Larsen et al., 2001). For instance, people may feel envy toward others’ success while still maintaining some empathy to meet relational norms (Deng et al., 2016). In online contexts, where social cues and normative pressures are weakened, relative deprivation may therefore heighten antagonistic emotions while eroding affiliative emotions. Therefore, we argue that relative deprivation, which simultaneously amplifies negative social emotions as well as erodes positive social emotions, increases vulnerability to cyber-ostracism.

Consistent with general strain theory, relative deprivation represents a strain involving perceived injustice that may elicit negative affect (Agnew, 2015; Wang et al., 2023). Attribution theory of emotion further suggests that individuals experiencing high relative deprivation tend to attribute others’ online success to social injustice, fueling their antagonistic feelings, such as anger and envy (Weiner, 1985; Zeng et al., 2023). These emotions may interfere with online interpersonal exchange. For instance, heightened jealousy often manifests online as antagonistic communication, which peers may perceive as unfriendly or socially inappropriate, thereby inciting overt exclusion (Pancani et al., 2023; Wang and Li, 2022). Indeed, prior studies have found that negative social emotion mediates the association between relative deprivation and online aggressive behavior (Huang et al., 2025; Zhu et al., 2024).

Importantly, the socioemotional consequences of relative deprivation are not limited to heightened distress; relative deprivation may also undermine positive social emotions that sustain affiliative exchange. In particular, individuals who feel relatively deprived tend to focus on their own disadvantaged position, unmet needs, and perceived unfairness. This self-focused appraisal may reduce the attentional and emotional resources available for considering others’ suffering or needs (Xu et al., 2023; Shah et al., 2012). Empirically, relative deprivation has been linked to lower gratitude (Yu et al., 2020) and lower empathy, which in turn, predicts reduced prosocial behavior (Xu et al., 2023). Because online ties are maintained largely through frequent low-cost signals of attention and warmth, diminished positive social emotions can undermine reciprocity and increase vulnerability to cyber-ostracism (Sels et al., 2021; van Kleef and Côté, 2022). Additionally, positive affect can function as a relational signal; when such signals are weak or absent, individuals may be perceived as less approachable or less valuable interaction partners, thus reducing others’ motivation to engage (Li et al., 2021; Santor and Yazbek, 2006). Together, social emotion may represent a critical psychological mechanism linking relative deprivation to cyber-ostracism. Thus, we present the following hypothesis on the basis of the literature reviewed above:

*H2*: Positive and negative social emotions mediate the relationship between relative deprivation and cyber-ostracism.

### 1.3 The moderating role of self-esteem

Although relative deprivation may influence individuals’ experiences of cyber-ostracism through positive and negative social emotions, not all individuals are affected to the same extent. Previous research suggests that the interaction between relative deprivation and individual traits, such as self-esteem, may jointly shape emotional and behavioral responses (Liao et al., 2024). *Self-esteem* is defined as an individual’s evaluation of their self-worth, perception of competence, and acceptance of the overall self (Rosenberg et al., 1995), and this plays a key role in emotional regulation and stress coping (Hatun and Türk Kurtça, 2025).

Numerous studies have shown that individuals with high self-esteem tend to possess stronger coping resources and are more capable of maintaining emotional stability in the face of adversity or stress (Li et al., 2023; Moksnes et al., 2010; Amestoy et al., 2023; Gao et al., 2024). The buffering model of self-esteem (Cast and Burke, 2002) further states that high self-esteem mitigates the emotional impact of negative events, whereas low self-esteem amplifies emotional distress during adversity. For example, Fang et al. (2021) found that self-esteem significantly moderated the relationship between stigma and psychological distress; hence, individuals with high self-esteem reported lower levels of distress when facing social stressors.

Moreover, self-esteem may influence how individuals cognitively and emotionally respond to relative deprivation. In particular, those with low self-esteem are more likely to focus on the disadvantageous aspects of their relative standing, leading to stronger feelings of inferiority and dissatisfaction with their circumstances (Blanton, 2013). In contrast, individuals with high self-esteem tend to interpret relative disadvantage as opportunities for growth rather than threats; thus, they are likely to engage in positive self-regulation strategies to maintain emotional balance (Vohs and Heatherton, 2004), thereby buffering emotional fluctuations triggered by perceived social injustice. High self-esteem may also facilitate more adaptive appraisal processes, enabling individuals to attribute negative outcomes to situational rather than personal causes, and this coping process further protects them from excessive emotional reactivity. In addition, self-esteem is significantly associated with greater positive social emotion and lower negative social emotion (Reitz, 2022; Zell and Johansson, 2024). These findings suggest that individuals with high self-esteem are better equipped to regulate their emotional states, enabling them to cope more effectively with the emotional consequences of relative deprivation and maintain psychological adjustment.

Beyond its role in shaping emotional responses, self-esteem may also moderate the direct association between relative deprivation and cyber-ostracism. When experiencing relative deprivation, individuals with low self-esteem may have fewer psychological resources for maintaining constructive and reciprocal online interactions. They may consequently respond with greater defensiveness, hostility, or interpersonal withdrawal, which can disrupt social exchange and increase the likelihood of being ignored or excluded. In contrast, individuals with high self-esteem may cope with relative deprivation more adaptively, maintain more stable interpersonal engagement, and avoid interaction patterns that contribute to social disengagement. Together, these processes suggest that self-esteem may weaken the association between relative deprivation and experiences of cyber-ostracism. Thus, we hypothesize the following on the basis of the literature reviewed above:

*H3a*: Self-esteem moderates the association between relative deprivation and positive social emotions.

H3b: Self-esteem moderates the association between relative deprivation and negative social emotions.

H3c: Self-esteem moderates the direct association between relative deprivation and cyber-ostracism.

### 1.4 The current study

**Figure 1:**
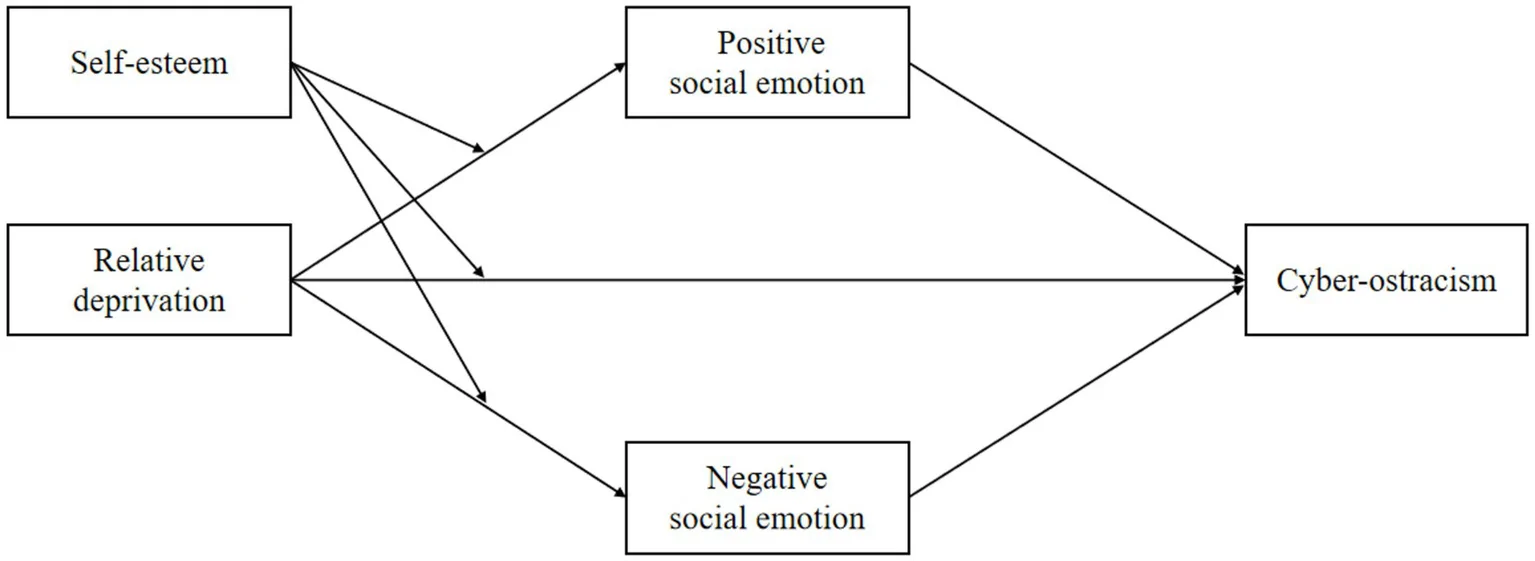
Proposed moderated dual mediation model.

Prior research has linked relative deprivation to offline ostracism and maladaptive online behavior (Jiang and Chen, 2020; Zhu et al., 2024), yet its association with cyber-ostracism remains insufficiently understood. Guided by general strain theory, the present study conceptualizes relative deprivation as perceived strain arising from unfavorable social comparison and integrates attribution theory of emotion and the buffering model of self-esteem to clarify the affective pathway and boundary condition of this process. Specifically, we propose a moderated parallel-mediation model (Figure 1) in which positive and negative social emotions mediate the association between relative deprivation and cyber-ostracism, while self-esteem moderates these pathways. These findings may provide theoretical support for future interventions aimed at mitigating the emotional consequences of relative deprivation and reducing vulnerability to cyber-ostracism.

## 2 Methods

### 2.1 Participants and procedures

Participants were undergraduate students recruited through convenience sampling from two universities in Guangzhou, Guangdong Province. Data were collected between August and October 2024. Prior to participation, participants were provided with detailed study information and informed about their right to withdraw from the study at any point. After obtaining informed consent, participants independently completed the online questionnaires on their smartphones via the online platform Questionnaire Star.^1^ All participants received course credits after completing the survey. This study was approved by the Research Ethical Committee of the first author’s institution.

A total of 1,587 questionnaires were collected. Following data screening, responses were excluded if the completion time was less than 180 s or if 10 or more consecutive identical responses were detected (Shi et al., 2025). After these exclusions, 1,508 valid responses were retained, resulting in a valid response rate of 95.02%. The final sample consisted of 653 females (43.3%) and 855 males (56.7%), with an average age of 19.43 (SD = 1.11; range = 18–21 years).

### 2.2 Measures

#### 2.2.1 Relative deprivation

We assessed relative deprivation with the Chinese version of the Adolescents’ Relative Deprivation Scale (Tian et al., 2021). This scale has 10 items measuring college students’ relative deprivation (e.g., “Compared to the people around me, I feel dissatisfied that I have fewer opportunities to develop my interests than they do.”), rated on a 5-point scale (from “1 = *Strongly disagree*” to “5 = *Strongly agree*”). Cronbach’s alpha was 0.85.

#### 2.2.2 Self-esteem

We measured self-esteem with the Chinese version of the Rosenberg Self-Esteem Scale (Wang et al., 2025; Rosenberg, 1965). This scale has 10 items measuring college students’ self-esteem (e.g., “I take a positive attitude toward myself”), rated on a 4-point scale (from “1 = *Strongly disagree*” to “4 = *Strongly agree*”). Cronbach’s alpha was 0.86.

#### 2.2.3 Social emotion

We measured social emotion with the 28-item Chinese version of the Social Emotion Scale (Yang et al., 2023). The scale includes two subscales, positive social emotion and negative social emotion, which have also been treated as distinguishable components of social emotion in subsequent empirical studies (Yang et al., 2024; Yang et al., 2026). The positive social emotion subscale includes 12 items reflecting social pride, social sympathy, and social gratitude, whereas the negative social emotion subscale includes 16 items reflecting social indifference, social complaint, social anxiety, and social restlessness. Following this scale structure, the present study used the two subscale scores in the main analyses. These items were rated on a 5-point scale (from “1 = *Extremely disagree*” to “5 = *Extremely agree*”). Higher mean scores represent higher levels of positive/negative social emotion. The Cronbach’s αs of the positive social emotion and negative social emotion were 0.91 and 0.85, respectively.

#### 2.2.4 Cyber-ostracism

We assessed cyber-ostracism with the Chinese version of the cyber-ostracism questionnaire (Tong, 2015; Xing and Kuo, 2024). This scale has 14 items measuring college students’ self-reported experiences of being ignored or excluded in online interactions in daily life (e.g., “I encountered problems and sought help online, but received no feedback”), rated on a 5-point scale (from “1 = *never*” to “5 = *always*”). These items mainly reflect passive online exclusion, such as online ignoring or nonresponse, rather than active exclusion, such as being removed from group chats or publicly blocked. Cronbach’s alpha was 0.93.

### 2.3 Data analysis

First, we conducted descriptive statistics, correlation analysis, and reliability analysis in SPSS 26.0. Second, we performed a mediation model to test the mediating effect of positive social emotion and negative social emotion between relative deprivation and cyber-ostracism in Mplus 8.3 (Muthén and Muthén, 1998–2017). Third, based on the mediation model, we included self-esteem as a moderator to test the moderating effect of self-esteem on the relation between relative deprivation and social emotion. Before constructing the interaction term, both the independent variable and the moderator were mean-centered. If the interacting effect between relative deprivation and self-esteem on social emotion was significant, we further investigated the simple slopes effect and the moderated mediating effects of social emotion. Across these models, age and gender were both included as covariates. Bootstrapping (*N* = 5,000) and its 95% confidence interval (CI) were conducted to judge the significance of (moderated) mediating effect. All questionnaire items were set as required responses on the online survey platform; therefore, no item-level missing data remained in the final analytic sample (Hayes, 2015).

Monte Carlo power analysis was conducted to estimate the statistical power for detecting indirect effects in the parallel mediation model (Schoemann et al., 2017). With a sample size of 1,508, the power to detect the indirect effect of positive social emotion was 1.00, while the power for the indirect effect of negative social emotion was 0.90. These results indicate that the study was well-powered to detect both indirect effects in the mediation model.

## 3 Results

### 3.1 Common method bias

Given that all variables were measured using self-report questionnaires, we examined the potential influence of common method variance (Podsakoff et al., 2003). Harman’s single-factor test was first conducted by entering all 62 items into an unrotated exploratory factor analysis. The analysis extracted 12 factors with eigenvalues greater than 1, and the first factor accounted for 21.53% of the total variance, below the conventional 40% criterion. We further conducted confirmatory factor analysis to compare the hypothesized five-factor structure, comprising relative deprivation, self-esteem, positive social emotion, negative social emotion, and cyber-ostracism, with a one-factor alternative in which all indicators loaded onto a single latent factor (Conway and Lance, 2010). The five-factor model showed acceptable fit, *χ*^2^/df = 3.72, TLI = 0.981, CFI = 0.985, RMSEA = 0.042, whereas the one-factor model fit the data poorly, *χ*^2^/df = 98.23, TLI = 0.311, CFI = 0.403, RMSEA = 0.254, ΔCFI = 0.582. These results suggest that common method variance was unlikely to seriously bias the present findings.

### 3.2 Descriptive statistics and bivariate correlation

The descriptive results were summarized in Table 1. As shown in Table 1, relative deprivation was negatively associated with positive social emotion (*r* = −0.24, *p* < 0.001), and positively associated with negative social emotion (*r* = 0.50, *p* < 0.001) as well as cyber-ostracism (*r* = 0.38, *p* < 0.001). Cyber-ostracism was negatively associated with positive social emotion (*r* = −0.23, *p* < 0.001) and positively associated with negative social emotion (*r* = 0.27, *p* < 0.001). Self-esteem was negatively associated with relative deprivation (*r* = −0.44, *p* < 0.001) as well as negative social emotion (*r* = −0.35, *p* < 0.001), and positively associated with positive social emotion (*r* = 0.32, *p* < 0.001).

**Table 1:** The means, standard deviations, reliabilities and correlations among the variables.

Variable	1	2	3	4	5	6	7
Key variables							
1. Relative deprivation							
2. Self-esteem	−0.44^***^						
3. Positive social emotion	−0.24^***^	0.32^***^					
4. Negative social emotion	0.50^***^	−0.35^***^	−0.23^***^				
5. Cyber-ostracism	0.38^***^	−0.30^***^	−0.23^***^	0.27^***^			
Covariates							
6. Age	0.00	−0.02	−0.06^*^	−0.03	0.03		
7. Gender	0.10^***^	−0.05	−0.23^***^	0.12^***^	0.18^***^	0.02	
*M*	2.68	2.89	4.23	2.67	1.80	19.43	43.30^a^
*SD*	0.61	0.45	0.58	0.56	0.64	1.11	
α	0.85	0.86	0.91	0.85	0.93	–	–

Gender: 1 = Male, 0 = Female; ^*^p < 0.05, ^***^p < 0.001.

a The percentage of female.

### 3.3 The mediating effect of social emotions between relative deprivation and cyber-ostracism

**Figure 2:**
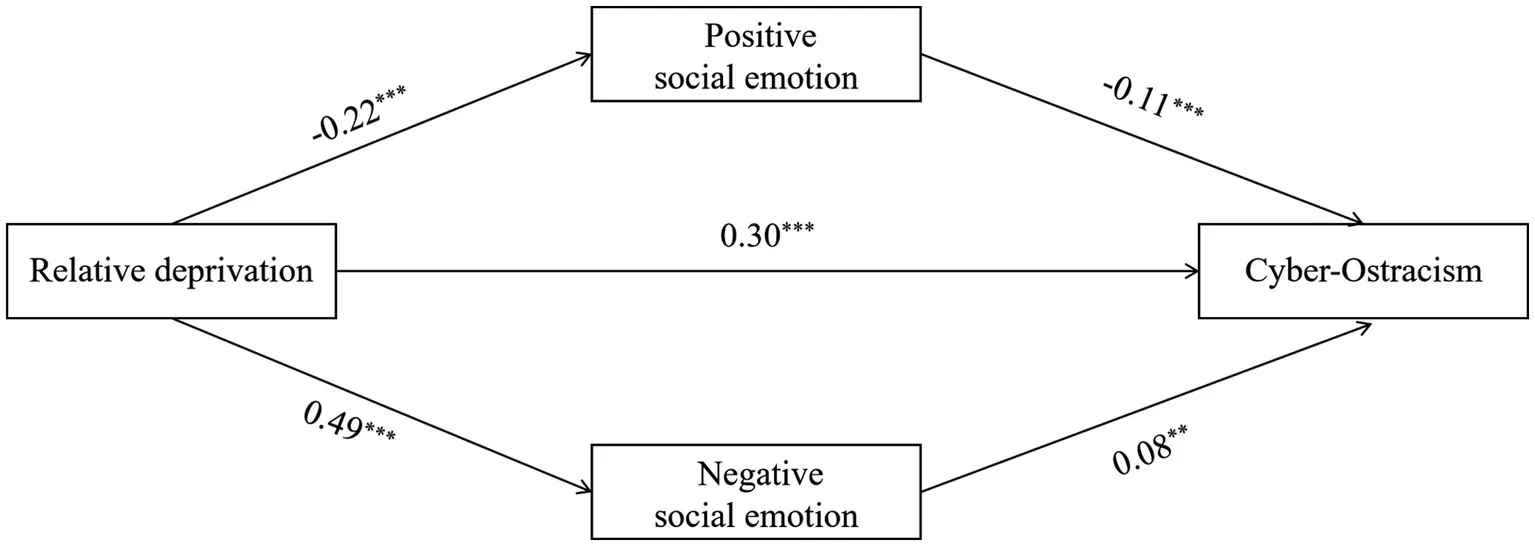
The mediating effect of social emotion in the relation between relative deprivation and cyber-ostracism. Standardized coefficients are reported. ^**^p < 0.01, ^***^p < 0.001.

As depicted in Figure 2, after controlling for age and gender, relative deprivation was negatively related to positive social emotion (*β* = −0.22, *SE* = 0.03, *p* < 0.001), and positively related to negative social emotion (*β* = 0.49, *SE* = 0.02, *p* < 0.001) as well as cyber-ostracism (*β* = 0.30, *SE* = 0.03, *p* < 0.001). Positive social emotion was negatively related to cyber-ostracism (*β* = −0.11, *SE* = 0.03, *p* < 0.001), and negative social emotion was positively related to cyber-ostracism (*β* = 0.08, *SE* = 0.03, *p* = 0.003). Results of mediation analyses showed that mediation effect of positive social emotion [*Indirect effect* = 0.03, *SE* = 0.01, 95% CI = (0.011, 0.039)] and negative social emotion were significant [*Indirect effect* = 0.04, *SE* = 0.01, 95% CI = (0.012, 0.069)] (see Table 2). The indirect effects through positive social emotion and negative social emotion accounted for 6.78 and 10.82% of the total effect, respectively, and the total indirect effect accounted for 17.60% of the total effect. The direct effect remained significant, supporting a partial mediation pattern.

**Table 2:** The mediating effect of social emotion in the relation between relative deprivation and cyber-ostracism.

Effect	Standardized effect	95% CI	SE
Total effect	0.37	[0.321, 0.409]	0.02
Direct effect	0.30	[0.247, 0.354]	0.03
Total indirect effect	0.06	[0.036, 0.095]	0.02
Path 1: Relative deprivation → Positive social emotion → Cyber-ostracism	0.03	[0.011, 0.039]	0.01
Path 2: Relative deprivation → Negative social emotion → Cyber-ostracism	0.04	[0.012, 0.069]	0.01

### 3.4 The moderating effect of self-esteem

**Figure 3:**
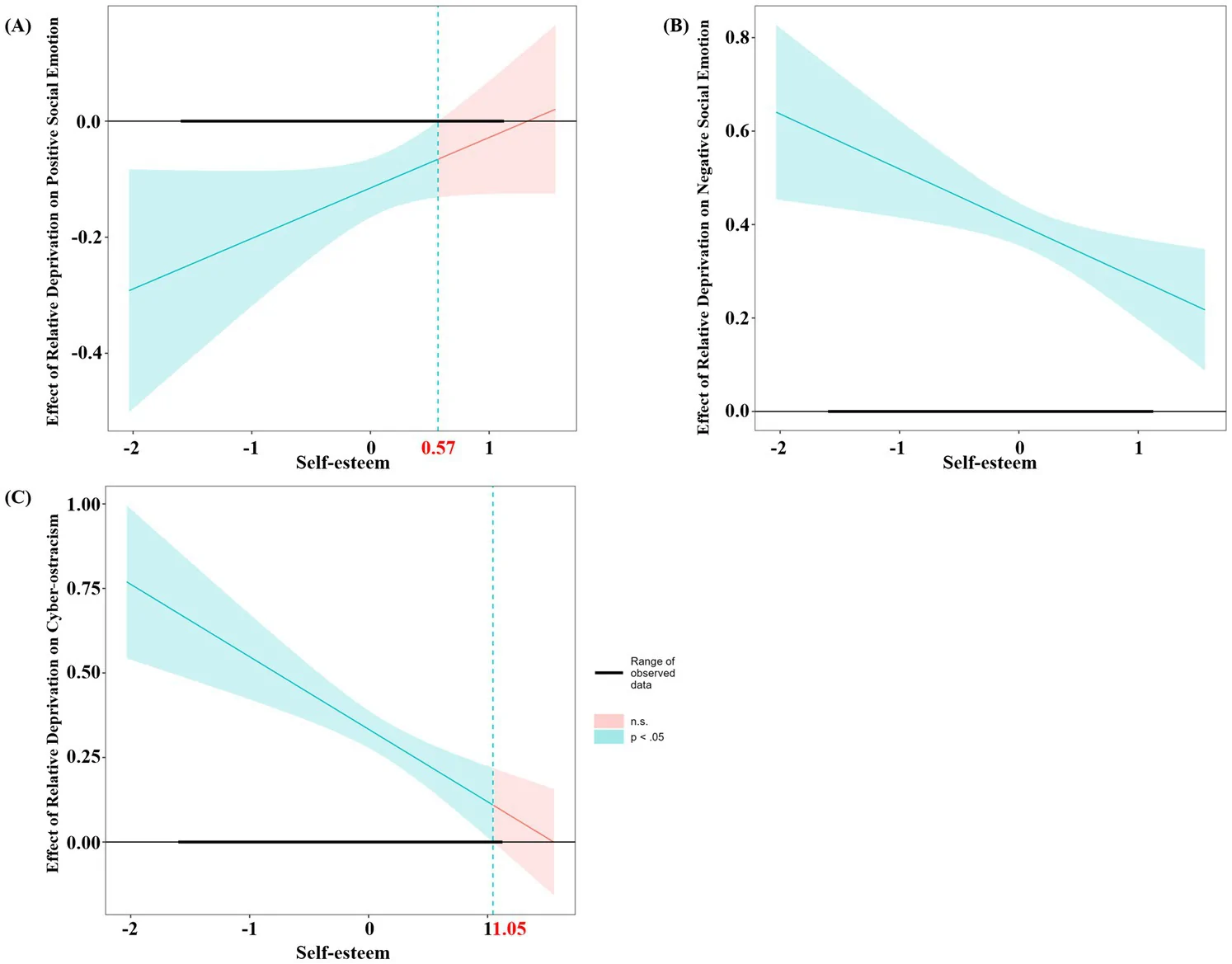
**(A)** The relation between relative deprivation and positive social emotion moderated by self-esteem; **(B)** The relation between relative deprivation and negative social emotion moderated by self-esteem; **(C)** The relation between relative deprivation and cyber-ostracism moderated by self-esteem.

As detailed in Table 3, the moderated mediating model fit the data well [RMSEA = 0.06 (90% CI = (0.049, 0.080)), CFI = 0.955, and SRMR = 0.03]. The results indicated that self-esteem moderated the impact of relative deprivation on positive social emotion (*β* = 0.05, *SE* = 0.02, *p* = 0.024), negative social emotion (*β* = −0.06, *SE* = 0.02, *p* = 0.004), and cyber-ostracism (*β* = −0.09, *SE* = 0.03, *p* = 0.001). After breaking the interaction effect, Johnson-Neyman analysis (see Figure 3) revealed that relative deprivation was negatively associated with positive social emotion when self-esteem was within the interval [−1.59, 0.57]. This pattern suggested that the negative impact of relative deprivation on positive social emotion was particularly salient among individuals with relatively lower self-esteem. When self-esteem was below 0.57, relative deprivation had a significantly negative effect on positive social emotion; when self-esteem was above 0.57, the effect of relative deprivation on positive social emotion was no longer significant. Regarding negative social emotion, JN results indicated that relative deprivation was positively associated with negative social emotion when self-esteem was within the interval [−1.59, 1.11]. As self-esteem increased, the effect of relative deprivation on negative social emotion gradually diminished. Regarding cyber-ostracism, JN results showed that relative deprivation was positively associated with cyber-ostracism when self-esteem was within the interval [−1.59, 1.05], which nearly encompassed the entire observed range of self-esteem [−1.59, 1.11].

**Table 3:** The moderated mediation model.

Predictor	Positive social emotion (*R*^2^ = 0.15)			Negative social emotion (*R*^2^ = 0.28)			Cyber-ostracism (*R*^2^ = 0.20)		
	*β*	*SE*	*p*	*β*	*SE*	*p*	*β*	*SE*	*p*
Covariates									
Age	−0.05	0.02	0.031	−0.04	0.02	0.105	0.02	0.02	0.383
Gender	−0.20	0.02	< 0.001	0.07	0.02	0.002	0.12	0.02	< 0.001
Key variables									
Relative deprivation	−0.12	0.03	< 0.001	0.44	0.03	< 0.001	0.28	0.03	< 0.001
Self-esteem	0.26	0.03	< 0.001	−0.17	0.03	< 0.001	−0.15	0.03	< 0.001
Relative deprivation × self-esteem	0.05	0.02	0.024	−0.06	0.02	0.004	−0.09	0.03	0.001
Positive social emotion							−0.08	0.03	0.009
Negative social emotion							0.05	0.03	0.063

Gender: 1 = Male, 0 = Female.

Moreover, Table 4 summarized the mediating effect of social emotion by the levels of self-esteem. The mediating effect of positive social emotion was stronger when the levels of self-esteem were low [*Indirect effect* = 0.01, *SE* = 0.01, 95% CI = (0.003, 0.025)] than when the levels of self-esteem were high [*Indirect effect* = 0.01, *SE* = 0.00, 95% CI = (0.001, 0.013)]. Similarly, the mediating effect of negative social emotion was stronger when the levels of self-esteem were low [*Indirect effect* = 0.03, *SE* = 0.01, 95% CI = (−0.001, 0.054)] than when the levels of self-esteem were high [*Indirect effect* = 0.02, *SE* = 0.01, 95% CI = (−0.001, 0.042)]. In the full moderated mediation model, the path from positive social emotion to cyber-ostracism remained significant (*β* = −0.08, *p* = 0.009), whereas the path from negative social emotion to cyber-ostracism was marginally significant (*β* = 0.05, *p* = 0.063). Overall, the moderated mediation model provided stronger support for the positive social emotion pathway.

**Table 4:** Conditional indirect effects and index of moderated mediation.

Condition	Indirect effect	SE	95% CI
Positive social emotion as dependent variable			
Low 1 (*M* − *SD*)	0.01	0.01	[0.003, 0.025]
High 1 (*M* + *SD*)	0.01	0.00	[0.001, 0.013]
Diff 1 = High 1 − Low 1	−0.01	0.00	[−0.015, −0.000]
Negative social emotion as dependent variable			
Low 2 (*M* − *SD*)	0.03	0.01	[−0.001, 0.054]
High 2 (*M* + *SD*)	0.02	0.01	[−0.001, 0.042]
Diff 2 = High 2 − Low 2	−0.01	0.00	[−0.016, 0.000]
Index of moderated mediation	−0.006	0.00	[−0.012, −0.002]

Furthermore, we computed the indices of moderated mediation to test whether these indirect effects depended on self-esteem. Self-esteem significantly moderated the indirect effect of relative deprivation on cyber-ostracism via positive social emotion [*β* = −0.003, *SE* = 0.002, 95% CI (−0.008, −0.000)], with this indirect effect weakening as self-esteem increased. The index was not significant for the negative social emotion pathway [*β* = −0.003, *SE* = 0.002, 95 %CI (−0.008, 0.000)]. The total index of moderated mediation was significant [*β* = −0.006, *SE* = 0.003, 95% CI (−0.012, −0.002)]. Overall, these results suggest that self-esteem moderated the indirect association between relative deprivation and cyber-ostracism, particularly through positive social emotion.

### 3.5 Sensitivity analysis

To address concerns about the robustness of the cross-sectional moderated mediation model, we conducted a random-subsample robustness check by re-estimating the model in a randomly selected subsample of 500 participants. As shown in Supplementary Table S1, the main regression paths were generally consistent with those observed in the full sample. Relative deprivation significantly predicted positive social emotion, negative social emotion, and cyber-ostracism. Self-esteem also significantly moderated the associations between relative deprivation and positive social emotion, negative social emotion, and cyber-ostracism.

As shown in Supplementary Table S2, the conditional indirect effects further indicated that the indirect effect via positive social emotion was significant at both low and high levels of self-esteem, and the difference between these two conditional indirect effects was significant. The index of moderated mediation for this pathway was also significant [*β* = −0.010, *SE* = 0.006, 95% CI = (−0.023, −0.001)]. In contrast, the conditional indirect effects via negative social emotion were not significant in the subsample, and the corresponding index of moderated mediation was also not significant [*β* = −0.014, *SE* = 0.009, 95% CI (−0.034, 0.001)]. Nevertheless, the total index of moderated mediation remained significant [*β* = −0.024, *SE* = 0.010, 95% CI = (−0.046, −0.007)]. Overall, the random-subsample analysis provided partial support for the robustness of the proposed model, with the positive social emotion pathway showing more stable evidence than the negative social emotion pathway.

## 4 Discussion

Guided by general strain theory, the present study examined how relative deprivation is associated with cyber-ostracism through positive and negative social emotions and whether self-esteem conditions these associations. Building on prior evidence linking relative deprivation to offline ostracism (Jiang and Chen, 2020), the present study found a positive association between relative deprivation and cyber-ostracism. This finding suggests that the exclusion-related implications of relative deprivation may extend to online contexts, where withdrawal, indifference, or hostility can disrupt reciprocal interaction and increase the likelihood of being ignored or excluded (Tao et al., 2023; Yuan et al., 2025). The two emotional pathways, however, differed in consistency. The indirect pathway through diminished positive social emotion remained supported across models, whereas evidence for the pathway through heightened negative social emotion weakened in the full moderated model. This pattern highlights the role of diminished positive social emotion in the association between relative deprivation and cyber-ostracism.

### 4.1 The mediating role of positive and negative social emotions

In the mediation model, relative deprivation was indirectly associated with cyber-ostracism through both lower positive social emotion and higher negative social emotion. This pattern is consistent with general strain theory, which suggests that perceived disadvantage can evoke socioemotional responses that shape interpersonal behavior (Agnew, 2015; Mummendey et al., 1999; Smith et al., 2012). Individuals who feel relatively deprived may experience stronger anger, resentment, or dissatisfaction, which can be expressed in online interactions through hostile communication, dismissive responses, or interpersonal withdrawal. These interaction patterns may disrupt reciprocity and increase the likelihood of being ignored or excluded online (Greitemeyer and Sagioglou, 2019a; Lin et al., 2024; Wang et al., 2023). This pathway may also be understood in terms of supportive relational resources. Social support can buffer individuals against stressful or exclusionary experiences, whereas low perceived support has been linked to greater ostracism-related risk (Yu et al., 2021). Thus, individuals with higher negative social emotion may have fewer resources to repair strained online interactions or recover from online ignoring and nonresponse, making cyber-ostracism more difficult to interrupt. However, the indirect pathway through negative social emotion was less consistent in the full moderated mediation model. This may reflect overlap between negative social emotion and unmeasured internalizing or exposure-related factors. Loneliness and social anxiety may heighten negative affect, while loneliness may also increase reliance on online interaction for social connection, thereby increasing opportunities to experience online ignoring, reduced feedback, or nonresponse (Nowland et al., 2018; Shannon et al., 2022). Therefore, the weaker negative social emotion pathway may partly reflect shared variance with loneliness, social anxiety, and online exposure rather than the absence of deprivation-related distress.

The pathway through lower positive social emotion suggests that relative deprivation may be associated with cyber-ostracism through the loss of affiliative socioemotional resources, not only through heightened negative reactions. Positive emotions serve important social functions by strengthening interpersonal bonds and shaping how others respond to one’ s emotional expressions (Sels et al., 2021; van Kleef and Lelieveld, 2022). In online interactions, where social connection is often maintained through brief reciprocal signals such as attention, replies, and support, reduced positive social emotion may make constructive exchange more difficult to sustain (Stsiampkouskaya et al., 2023). This interpretation is consistent with evidence that relative deprivation can reduce empathy and prosocial behavior (Xu et al., 2023). Thus, the present findings extend prior work by identifying diminished positive social emotion as a pathway linking relative deprivation to cyber-ostracism.

### 4.2 The moderating role of self-esteem

Self-esteem moderated the associations of relative deprivation with positive social emotion, negative social emotion, and cyber-ostracism. Specifically, the negative association between relative deprivation and positive social emotion was weaker among individuals with higher self-esteem, and the positive associations of relative deprivation with negative social emotion and cyber-ostracism were also attenuated at higher levels of self-esteem. These findings support the buffering role of self-esteem in deprivation-related online experiences, extending prior evidence that self-esteem helps individuals cope with stress and maintain better emotional functioning (Cast and Burke, 2002; Jiang et al., 2025; Reitz, 2022; Zell and Johansson, 2024). This buffering pattern may reflect the role of self-esteem as a source of self-evaluative and relational security. From a sociometer perspective, self-esteem reflects perceived relational value, and individuals with higher self-esteem may be less likely to experience unfavorable social experiences as personal devaluation or threatened belonging (Leary and Baumeister, 2000). Thus, higher self-esteem may help individuals preserve positive social emotion, reduce negative emotional reactivity, and maintain more stable online engagement when facing deprivation-related strain. Additionally, self-esteem may weaken the link between relative deprivation and cyber-ostracism by shaping how ambiguous online interactions are interpreted. Individuals with lower self-esteem may be more vulnerable to ego threat during social comparison and more likely to interpret ambiguous interpersonal cues in rejection-oriented ways (Downey and Feldman, 1996; Shi et al., 2025).

### 4.3 Limitations and future research

The present study has several limitations that should be acknowledged. First, the use of a cross-sectional design limits conclusions about temporal ordering and causal inference. Although the present model conceptualized relative deprivation as a potential antecedent of cyber-ostracism, the association may be reciprocal rather than strictly unidirectional. Being ignored or excluded online may heighten feelings of relative disadvantage, as ostracism threatens fundamental needs for belonging, self-esteem, control, and meaningful existence (Williams, 2009), and perceived ostracism has been shown to increase relative deprivation (Jiang and Chen, 2020). Future research should use longitudinal designs, such as cross-lagged panel models or daily diary designs, to examine the temporal dynamics between these constructs. Experimental studies that manipulate relative deprivation or online exclusion cues could also help clarify causal processes.

Second, the assessment instruments primarily relied on self-report measures, which are susceptible to reporting bias, social desirability bias, and common method variance. Future studies could incorporate more objective indicators of online exclusion, as well as multi-source data such as peer reports, behavioral observations, experience-sampling assessments, or digital trace records.

Third, the generalizability of the findings may be limited by the cultural, regional, and demographic characteristics of the sample. Participants were recruited from Guangdong Province, China, and only age and gender were assessed as demographic covariates. Thus, the present study could not examine whether the proposed model varies by socioeconomic status, academic major, year of study, or urban/rural background. The associations among relative deprivation, self-esteem, and cyber-ostracism may also operate differently across cultural contexts. In more collectivistic contexts, concerns about relational harmony, social belonging, and interpersonal evaluation may shape how individuals experience deprivation and online exclusion, whereas in more individualistic contexts, self-esteem and personal autonomy may play different roles (Klein et al., 2024; Uskul and Over, 2017). Future research should examine whether the present model holds across different cultural settings, socioeconomic backgrounds, and developmental groups.

Fourth, several potentially relevant variables were not measured in the present study. Social comparison orientation may contribute to the formation of relative deprivation, whereas loneliness, social anxiety, and internet-use duration may influence both online interaction patterns and reported experiences of cyber-ostracism (Gibbons and Buunk, 1999; Kardefelt-Winther, 2014; Nowland et al., 2018; Shannon et al., 2022). Because these variables may be associated with both the predictors and outcomes in the present model, the observed direct, indirect, and moderated associations may partly reflect unmeasured confounding. Although the random-subsample analysis provided partial evidence for the stability of the model, it cannot rule out omitted-variable bias. Future research should include these variables as covariates or competing mechanisms and use longitudinal, experience-sampling, or experimental designs to examine the robustness of the present model. In addition, future studies may consider psychological resources beyond self-esteem, such as meaning in life, resilience, and positive childhood experiences, which have been linked to maladaptive online experiences and psychosocial outcomes (Çiçek et al., 2025; Ünsal et al., 2025; Egan et al., 2024; Zhu et al., 2025).

### 4.4 Implications

The present findings suggest practical implications at the individual, educational, and platform levels. At the individual level, support should focus on how students respond to unfavorable social comparison online. Students who frequently compare themselves with idealized online self-presentations may benefit from training in cognitive reappraisal, self-affirmation, and reflective social media use (Cohen and Sherman, 2014; Gross, 2015). These strategies may help them recognize the selective nature of online self-presentation, reduce deprivation-related emotional reactions, and maintain more constructive online engagement.

At the educational level, schools and counseling services should address both deprivation-related strain and its emotional consequences. In addition to reducing negative emotions such as anger, resentment, and hostility, intervention programs may help students preserve positive social emotions, including gratitude, empathy, and warmth. Strategies such as gratitude exercises, empathy training, emotional reappraisal, strength-based feedback, and supportive peer activities may help students maintain constructive engagement and more stable self-evaluations when facing perceived disadvantage (Dickens, 2017; Xu et al., 2023).

At the platform level, online communities and campus-based digital platforms could reduce conditions that intensify upward comparison and social exclusion. For example, platforms may reduce the visibility of popularity metrics, diversify recommendation content to avoid repeated exposure to idealized achievements, and provide moderation tools that help identify posts or users repeatedly left without response in group interactions (Nesi and Prinstein, 2015; Yan et al., 2025; Yuan et al., 2025). These design strategies may help create online environments that are less comparison-driven and more responsive to users’ social participation.

## 5 Conclusion

Guided by general strain theory, this study employed a moderated parallel mediation model to examine the association between relative deprivation and cyber-ostracism in digital social contexts. By integrating positive and negative social–emotional pathways with a self-esteem–based buffering framework, the findings offer a more integrated understanding of how perceived disadvantage is linked to reported experiences of cyber-ostracism.

Furthermore, three key conclusions emerge. First, relative deprivation was positively associated with cyber-ostracism, suggesting that perceived disadvantage may be an important psychosocial correlate of online exclusion experiences. Second, positive and negative social emotions served as parallel mediating pathways in this association. Third, self-esteem moderates the emotional consequences of relative deprivation, attenuating negative social emotion while helping to maintain positive social emotion. Therefore, self-esteem functions as a protective psychological resource.

Taken together, the abovementioned findings advance theoretical insights into the interplay between relative deprivation, emotion, and online social outcomes. Furthermore, they provide a foundation for future prevention and intervention efforts aimed at reducing vulnerability to cyber-ostracism.

## Data Availability

The raw data supporting the conclusions of this article are included in the article/Supplementary material. Further inquiries can be directed to the corresponding author.

## Footnotes

1
https://www.wjx.cn/
