# Supplementary material for: The association between relative deprivation and cyber-ostracism: the mediating role of social emotions and the moderating role of self-esteem
Source: Front Psychol. 2026 Aug 6;17:1834878. doi: 10.3389/fpsyg.2026.1834878 (PMC13490128; doi:10.3389/fpsyg.2026.1834878)
Supplement: Supplementary file 1 [file Table_1.DOCX]

Supplementary Material

**Supplemental Table 1.** The moderated mediation model.

|  | Positive social emotion  (*R*^2^ = 0.21) | | |  | Negative social emotion  (*R*^2^ = 0.30) | | |  | Cyber-ostracism  (*R*^2^ = 0.28) | | |
| --- | --- | --- | --- | --- | --- | --- | --- | --- | --- | --- | --- |
|  | *β* | *SE* | *p* |  | *β* | *SE* | *p* |  | *β* | *SE* | *p* |
| **Covariates** |  |  |  |  |  |  |  |  |  |  |  |
| Age | -0.07 | 0.04 | 0.110 |  | -0.02 | 0.04 | 0.620 |  | 0.03 | 0.04 | 0.458 |
| Gender | -0.24 | 0.04 | < 0.001 |  | 0.05 | 0.04 | 0.175 |  | 0.06 | 0.04 | 0.107 |
| **Key variables** |  |  |  |  |  |  |  |  |  |  |  |
| Relative deprivation | -0.22 | 0.05 | < 0.001 |  | 0.44 | 0.04 | < 0.001 |  | 0.29 | 0.05 | < 0.001 |
| Self-esteem | 0.23 | 0.05 | < 0.001 |  | -0.21 | 0.04 | < 0.001 |  | -0.21 | 0.05 | < 0.001 |
| Relative deprivation ×  Self-esteem | 0.10 | 0.04 | 0.014 |  | -0.18 | 0.04 | < 0.001 |  | -0.13 | 0.04 | 0.002 |
| Positive social emotion |  |  |  |  |  |  |  |  | -0.11 | 0.04 | 0.013 |
| Negative social emotion |  |  |  |  |  |  |  |  | 0.09 | 0.05 | 0.051 |

*Note*. Gender: 1 = Male, 0 = Female.

**Supplemental Table 2.** Conditional indirect effects and index of moderated mediation.

|  | *Indirect effect* | *SE* | 95% CI |
| --- | --- | --- | --- |
| **Positive social emotion as dependent variable** |  |  |  |
| Low 1 (*M* - *SD*) | 0.03 | 0.02 | [0.002, 0.068] |
| High 1 (*M* + *SD*) | 0.01 | 0.01 | [0.001, 0.031] |
| Diff 1 = High 1 - Low 1 | -0.02 | 0.01 | [-0.046, -0.001] |
|  |  |  |  |
| **Negative social emotion as dependent variable** |  |  |  |
| Low 2 (*M* - *SD*) | 0.05 | 0.03 | [-0.003, 0.116] |
| High 2 (*M* + *SD*) | 0.03 | 0.01 | [-0.002, 0.056] |
| Diff 2 = High 2 - Low 2 | -0.03 | 0.02 | [-0.068, 0.002] |
|  |  |  |  |
| **Index of moderated mediation** | -0.024 | 0.01 | [-0.046, -0.007] |
